# A Cross-Sectional Analysis of Musculoskeletal and Functional Outcomes in Patients with Pituitary Disorders: Frailty and Sarcopenia

**DOI:** 10.3390/jcm15051835

**Published:** 2026-02-27

**Authors:** Kader Ugur, Mustafa Gur, Mithat Mızrak, Hasan Eryesil, Abdulvahap Hohluoglu, Hakan Artas, Kenan Bozbay, Burak Oz, Ahmet Karatas, Elif Emre, Mustafa Ata Aydin, İlknur Zeynep Acartürk, İbrahim Akdeniz, Suleyman Serdar Koca, Suleyman Aydın, Do-Youn Lee

**Affiliations:** 1 Department of Internal Medicine (Endocrinology and Metabolism Diseases), School of Medicine, Firat University, 23119 Elazig, Turkey; kaderaksoy06@hotmail.com (K.U.); mithat.mizrak@gmail.com (M.M.); 2 Department of Rheumatology, Faculty of Medicine, Firat University, 23119 Elazig, Turkey; m.gur@firat.edu.tr (M.G.); boz@firat.edu.tr (B.O.); drakaratas@yahoo.com (A.K.); kocassk@yahoo.com (S.S.K.); 3 Department of Radiology, School of Medicine, Firat University, 23119 Elazig, Turkey; hsneryesil@gmail.com (H.E.); hakanartas@yahoo.com (H.A.); 4 Department of Internal Medicine (Gastroenterology), School of Medicine, Firat University, 23119 Elazig, Turkey; vahap.hohluoglu@gmail.com; 5 School of Physical Education and Sports, Dicle University, 21280 Diyarbakir, Turkey; kenanbozbay21@gmail.com; 6 Department of Anatomy, School of Medicine, Firat University, 23119 Elazig, Turkey; eemre@firat.edu.tr; 7 School of Medicine, Gazi University, 06570 Ankara, Turkey; drataaydin@gmail.com; 8 Department of İnternal Medicine, Samsun Gazi State Hospital, 55070 Samsun, Turkey; izkilic@hotmail.com; 9 Department of Pediatric Surgery, School of Medicine, Firat University, 23119 Elazig, Turkey; iakdeniz@firat.edu.tr; 10 Firat Hormones Research Group, Department of Medical Biochemistry and Clinical Biochemistry, School of Medicine, Firat University, 23119 Elazig, Turkey; 11 College of General Education, Kookmin University, Seoul 02707, Republic of Korea

**Keywords:** acromegaly, frailty, hypopituitarism, muscle strength, sarcopenia

## Abstract

**Background/Objectives**: The purpose of this study was to compare the prevalence of sarcopenia and frailty in patients with acromegaly and hypopituitarism to healthy controls. Methods: This descriptive, comparative study included 32 patients with acromegaly, 24 patients with hypopituitarism, and 28 healthy volunteers who had undergone abdominal computed tomography (CT) within a month of their presentation at the endocrinology outpatient clinic between October 2023 and October 2024. The Tilburg Frailty Indicator was used to measure frailty. Sarcopenia was assessed using a dynamometer to measure handgrip strength and a CT-derived skeletal muscle index (SMI) at the L3 spinal level to measure muscle mass. **Results**: Both the hypopituitarism (7.5 ± 2.8) and acromegaly (7 ± 2.7) groups had substantially greater frailty ratings than the controls (4.8 ± 2.9) (*p* = 0.015). Frailty prevalence was 43% in the control group, 75% in acromegaly, and 83% in hypopituitarism (*p* = 0.019). The hypopituitarism group’s muscle strength (20.8 ± 7.4 kg) was substantially lower than that of the acromegaly (37.5 ± 14.3 kg) and control groups (36.4 ± 9.6 kg) (*p* < 0.001). Patients with hypopituitarism had substantially lower SMI values (45.1 ± 11.3 cm^2^/m^2^) than those with acromegaly (53 ± 8.9 cm^2^/m^2^) (*p* = 0.04). A total of 50% of the control group, 9% of those with acromegaly, and 54% of those with hypopituitarism had probable or confirmed sarcopenia (*p* < 0.001). **Conclusions**: This study shows that frailty is more prevalent in patients with acromegaly and hypopituitarism than in healthy controls, with sarcopenia being especially noticeable in hypopituitarism. After adjusting for age and sex, the association with frailty remained significant for hypopituitarism (OR = 5.24, *p* = 0.021) while acromegaly showed a borderline trend (OR = 3.00, *p* = 0.076). These results imply that pituitary hormones might contribute to maintaining the functional ability and integrity of the musculoskeletal system. Further research in prospective studies using population-based controls is necessary to examine screening for frailty and sarcopenia in patients with pituitary diseases.

## 1. Introduction

Frailty is a condition characterized by loss of biological reserves in multiple systems, homeostatic dysfunction, and increased sensitivity to stress [1]. While frailty is closely related to the aging process that leads to physiological decline, this decline is accelerated in frailty [2]. Adverse outcomes such as falls, hospitalization, and mortality are more likely to occur in frail patients [3]. Muscle strength and functional ability may deteriorate with age due to physiological and anatomical changes. The incidence of geriatric syndromes like frailty and sarcopenia rises concurrently with this process; frailty in particular is closely linked to poor long-term health outcomes [4,5,6]. Frailty is potentially preventable. Therefore, strategies aimed at preventing and slowing its progression are critically important [7]. However, by causing muscle loss and a reduction in functional ability, endocrine function abnormalities, especially those affecting anabolic processes, may contribute to frailty [8].

The endocrine system plays a significant role in frailty due to its complex relationship with the brain, immune system, and musculoskeletal system. The hypothalamic–pituitary axis plays a crucial role in the pathogenesis of frailty through the regulation of glucocorticoid secretion, insulin-like growth factor 1 (IGF-1), and androgen production [2]. By negatively impacting muscle regeneration, inflammatory control, and protein synthesis pathways, hypothalamic–pituitary axis dysfunction, which is crucial for preserving skeletal muscle mass and function, may exacerbate losses associated with sarcopenia [9,10]. Sarcopenia, a skeletal muscle condition marked by a progressive decrease in muscle mass and function as one ages, is closely linked to this process of muscle loss. Individuals and different muscle groups may have varying degrees of sarcopenia. This diversity makes it more difficult to identify appropriate management methods in addition to complicating the clinical assessment process [11,12].

Sarcopenia and frailty often coexist. As a result, a more thorough understanding of the frailty profile linked to unfavorable outcomes like falls, disability, and hospitalization may result from their combined evaluation [13]. Anabolic signaling may be weakened by imbalances in the GH/IGF-1 axis-related pathways linked to sarcopenia and frailty, which could lead to muscle loss and functional deterioration [14,15,16,17]. Skeletal muscle can be impacted by both GH’s direct signaling and IGF-1’s indirect signaling within this axis. Acromegaly is a well-known example of a pituitary illness that exhibits excess along this axis, which may offer a suitable clinical framework for investigating muscle involvement [18,19].

Acromegaly is a rare disease characterized by excessive secretion of growth hormone and its peripheral target hormone IGF-1 [20]. Acromegaly presents a paradoxical situation where, despite elevated anabolic hormone levels, patients may experience muscle dysfunction due to altered muscle fiber composition, connective tissue proliferation, and arthropathy [21]. Recent studies have identified qualitative muscle changes in acromegaly, including increased intramuscular fat deposition and reduced muscle quality despite preserved or increased muscle mass [22]. On the other hand, hypopituitarism is defined by pituitary hormone deficits, which may further impair skeletal muscle maintenance and regeneration, whereas acromegaly involves an excess of GH/IGF-1 [23,24,25].

In hypopituitarism, deficiencies in growth hormone (GH), gonadotropins, and other pituitary hormones can lead to reduced protein synthesis, impaired muscle regeneration, and increased muscle catabolism [26]. GH deficiency specifically results in decreased IGF-1 levels, which are essential for maintaining muscle mass and strength throughout life [27]. Additionally, secondary hypogonadism contributes to muscle wasting through reduced anabolic effects of sex hormones on skeletal muscle [28].

The prevalence and clinical characteristics of frailty and sarcopenia in people with pituitary diseases are not well documented, despite the fact that pituitary hormones are crucial to musculoskeletal health. In this cross-sectional investigation, we combined standardized frailty tests, muscle strength measurements, and CT-derived muscle mass indicators to examine the prevalence of frailty and sarcopenia in patients with hypopituitarism and acromegaly vs. healthy controls.

## 2. Materials and Methods

### 2.1. Study Design and Participants

This cross-sectional comparative study was conducted at the Endocrinology and Metabolism Diseases outpatient clinic of Firat University Hospital between October 2023 and October 2024. The study protocol was approved by the Firat University Ethics Committee (14714: 2023/13-01) and conducted in accordance with the Declaration of Helsinki. Before being enrolled, each subject gave written informed consent.

### 2.2. Sample Size Calculation

To calculate the sample size, G*Power software version 3.1.9.7 (Heinrich-Heine-Universität Düsseldorf, Düsseldorf, Germany) was used. The minimal sample size was determined to be *n* = 21–23 per group in order to detect clinically significant differences in frailty scores, based on prior research demonstrating moderate to large effect sizes (Cohen’s d = 0.6–0.8) for frailty differences in endocrine disorders [7], with α = 0.05 and power = 0.80. It was planned to recruit approximately 25–30 participants per group to allow for any dropouts and guarantee sufficient power for secondary analyses.

### 2.3. Study Population

This study comprised three groups: (1) patients with hypopituitarism (*n* = 24), (2) patients with acromegaly (*n* = 32), and (3) healthy control subjects (*n* = 28). Hypopituitarism was defined as the presence of at least two anterior pituitary hormone deficiencies confirmed by dynamic endocrine testing. The diagnosis of acromegaly was established based on elevated insulin-like growth factor 1 (IGF-1) levels and the absence of growth hormone (GH) suppression during an oral glucose tolerance test. Musculoskeletal complications are common in these patient populations and are primarily attributed to hormonal dysregulation secondary to pituitary dysfunction. Such endocrine disturbances may lead to decreased muscle mass, reduced bone mineral density, and impaired muscle strength. Moreover, the high prevalence of physical inactivity, chronic systemic comorbidities, and metabolic alterations further contributes to the frequency and severity of musculoskeletal impairment in these individuals.

Hospital employees and community volunteers without a history of endocrine, neuromuscular, or chronic inflammatory illnesses were chosen as controls. At inclusion, none of the controls had any recorded comorbidities and had undergone abdominal CT to assess nephrolithiasis.

**Figure 1:**
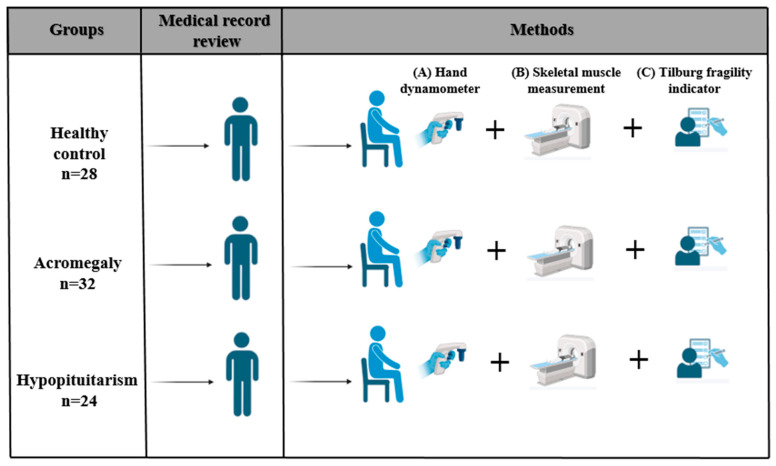
The study design is represented schematically. This study was carried out in three steps: (1) recruiting participants and classifying them into cohorts with hypopituitarism, acromegaly, and healthy controls; (2) measuring handgrip strength, evaluating skeletal muscle mass using the L3-level CT-derived skeletal muscle index (SMI), and assessing frailty using the Tilburg Frailty Indicator; and (3) statistical analysis comparing the results of sarcopenia and frailty across groups.

Every participant, who ranged in age from 18 to 75, had abdomen CT imaging performed for clinical reasons unrelated to musculoskeletal problems within a month after enrollment. Participants were excluded if they had severe organ failure, active malignancy, neuromuscular disorders unrelated to pituitary pathology, pituitary tumors, psychological disorders, a history of surgery within the preceding three months, or an inability to perform grip strength testing. As shown in Figure 1, the study design was divided into three phases.

### 2.4. Clinical Assessment

We collected demographic data, medical history, and current medications through patient interviews and medical records review. Disease duration was calculated from the date of diagnosis. Comorbidities and treatment history were systematically documented.

### 2.5. Assessment of Frailty

Frailty assessment was performed using the Tilburg Frailty Indicator. This scale consists of 15 questions, including physical (8 items), psychological (4 items), and social (3 items) sections. The total score ranges from 0 to 15, with higher scores indicating more severe frailty. A total score of 5 or higher is recommended for classifying a patient as frail. The Turkish validity and reliability of this scale were established by Topcu and colleagues [29].

### 2.6. Assessment of Sarcopenia

Handgrip strength and muscle mass measurement by computed tomography (CT) were used to evaluate patients for sarcopenia. Those with low muscle strength were considered to have “probable sarcopenia,” while those with both low muscle strength and low muscle mass were considered to have “confirmed sarcopenia” [30].

### 2.7. Muscle Strength Measurement

The standard grip strength of study participants was measured using a hand dynamometer (Model EH101, Zhongshan Camry Electronic Co., Ltd., Zhongshan, China). Measurements were taken in the standardized test position established by the American Hand Therapists Association, with the patient sitting upright in a chair without arm support, the shoulder in adduction, elbow at 90° flexion, forearm in neutral position, wrist in 0–30° extension, and 0–15° ulnar deviation. During the assessment, individuals were asked to squeeze with all their strength and then completely relax. This procedure was performed three times with the dominant hand, and the average of these values was recorded in kg/force. Normal grip strength values were determined as >27 kg for men and >16 kg for women [31].

### 2.8. Muscle Mass Measurement

A single axial slice (slice thickness: 1.25 mm) of abdominal CT images was used to calculate the cross-sectional area of the abdomen skeletal muscles at the L3 mid-vertebral level (cm^2^). Muscle segmentation was performed using [software name Horos v3.3.6 medical image software]. Skeletal muscle tissue was identified using Hounsfield unit (HU) thresholds of −29 to +150 HU. The muscles included in the segmentation were the psoas, erector spinae, quadratus lumborum, transversus abdominis, internal and external obliques, and rectus abdominis. All measurements were performed by a single trained radiologist (H.A.) who was blinded to group allocation. The skeletal muscle index (SMI) was calculated by dividing the total cross-sectional area by height squared (cm^2^/m^2^). Normal values were accepted as >52.4 cm^2^/m^2^ for men and >38.5 cm^2^/m^2^ for women [32].

### 2.9. Statistical Analysis

SPSS version 26.0 (IBM, New York, NY, USA) were used for statistical studies. Depending on the data distribution, descriptive statistics were displayed as means with standard deviations or medians for continuous variables and frequencies and percentages for categorical variables. The Shapiro–Wilk test was used to determine normality. Categorical variables were compared between groups using the chi-square test. For continuous variables, one-way ANOVA was employed when the data were normally distributed, whereas the Kruskal–Wallis test was applied for non-normally distributed data. Post hoc comparisons were conducted using Tukey’s test or Tamhane’s T2 test, depending on the homogeneity of variances. To adjust for potential confounders, binary logistic regression analyses were performed for frailty (TFI ≥ 5) and sarcopenia (probable or confirmed) as dependent variables, with group as the main predictor and adjustment for age and sex. Results were expressed as odds ratios (ORs) with 95% confidence intervals (CIs). After controlling for age and sex, continuous variables (TFI score, handgrip strength, and SMI) were compared between groups using analysis of covariance (ANCOVA). Cohen’s d effect sizes were computed for pairwise group comparisons of continuous outcomes, along with 95% confidence intervals. Due to collinearity with group assignment, comorbidity was excluded as a covariate in the primary models because the control group had no known comorbidities. A two-tailed *p*-value less than 0.05 was considered statistically significant.

## 3. Results

A total of 84 individuals were included in the study: 24 patients with hypopituitarism, 32 patients with acromegaly, and 28 healthy controls. The demographic characteristics of the groups are presented in Table 1. The mean ages of the groups were comparable (*p* = 0.99). Gender and education level distributions were not significantly different across the groups (*p* = 0.29 and *p* = 0.35, respectively). However, marital status differed significantly, with a lower proportion of married individuals in the acromegaly group compared to controls (*p* = 0.009). The mean disease duration was similar between the acromegaly and hypopituitarism groups (*p* = 0.54).

### 3.1. Medica, History, Treatment, and Comorbidities

Surgical management differed markedly between the two patient groups. The majority of acromegaly patients (87.5%) had undergone transsphenoidal surgery, compared to only half (50.0%) of hypopituitarism patients (*p* = 0.003). Radiotherapy was employed less frequently, with similar rates between groups (12.5% in acromegaly vs. 8.3% in hypopituitarism, *p* = 0.68). Comorbid conditions were prevalent in both groups, though more common in acromegaly patients (75.0%) than those with hypopituitarism (58.3%), though this difference was not statistically significant (*p* = 0.19). Treatment history and comorbid conditions for the patient groups are presented in Table 2. The control group had no documented comorbidities, and all underwent CT for nephrolithiasis evaluation, as described in Section 2.3.

### 3.2. Current Medical Treatment

Medication profiles clearly reflected the underlying pathophysiology of each condition. In the hypopituitarism group, hormone replacement therapy was nearly universal, with 95.8% receiving glucocorticoid replacement (hydrocortisone or prednisolone) and 79.2% requiring levothyroxine for central hypothyroidism. Sex hormone replacement was prescribed in 25.0% of hypopituitarism patients. Desmopressin was used in 16.7% for diabetes insipidus. Notably, only one patient (4.2%) received growth hormone replacement therapy, highlighting the limited availability of this treatment for adult GH deficiency in our setting. For acromegaly management, 62.5% of patients were receiving somatostatin analog therapy. Dopamine agonist therapy with cabergoline was used in 9.4% of acromegaly patients and 12.5% of hypopituitarism patients.

### 3.3. Frailty Scores and Frailty Status

The Tilburg Frailty Indicator scores are summarized in Table 3. One-way ANOVA followed by Tukey’s post hoc test revealed that frailty scores were significantly higher in both the hypopituitarism (mean ± SD: 7.5 ± 2.8) and acromegaly groups (7.0 ± 2.7) compared to controls (4.8 ± 2.9) (*p* = 0.01 and *p* = 0.04, respectively). No significant difference was found between the acromegaly and hypopituitarism groups (*p* = 0.75). Consistently, the prevalence of frailty was significantly higher in the hypopituitarism (83%) and acromegaly (75%) groups compared to controls (43%) (*p* = 0.019).

### 3.4. Muscle Strength and Muscle Mass Results

Muscle function parameters are also shown in Table 3. Muscle strength differed significantly among the groups according to Tamhane’s T2 post hoc test. The hypopituitarism group had significantly lower muscle strength (20.8 ± 7.4 kg) compared with both the acromegaly (37.5 ± 14.3 kg) and control groups (36.4 ± 9.6 kg) (*p* < 0.001). No difference was observed between the acromegaly and control groups (*p* = 0.98).

Regarding the skeletal muscle index (SMI), Tukey’s post hoc test showed that patients with hypopituitarism (45.1 ± 11.3 cm^2^/m^2^) had significantly lower values compared with those with acromegaly (53.0 ± 8.9 cm^2^/m^2^) (*p* = 0.04). No significant differences were found between the control group and either the acromegaly (*p* = 0.98) or hypopituitarism (*p* = 0.15) groups.

### 3.5. Sarcopenia Prevalence

The prevalence of sarcopenia (probable or confirmed) was significantly higher in the hypopituitarism (54%) and control groups (50%) compared to the acromegaly group (9%) (*p* < 0.001). Further classification of sarcopenia revealed that 42% of hypopituitarism, 9% of acromegaly, and 54% of control participants had probable sarcopenia (low muscle strength only), whereas the hypopituitarism group (12%, *n* = 3) confirmed sarcopenia (low muscle strength plus low muscle mass). Neither the control group nor the acromegaly participants satisfied the requirements for confirmed sarcopenia.

### 3.6. Multivariable Analysis

To account for potential confounders, binary logistic regression analyses were performed with adjustment for age and sex. For frailty (TFI ≥ 5), hypopituitarism was independently associated with significantly higher odds of frailty compared to controls (OR = 5.24, 95% CI: 1.28–21.45, *p* = 0.021). The association between acromegaly and frailty showed a trend toward significance (OR = 3.00, 95% CI: 0.89–10.10, *p* = 0.076). Female sex was also a significant independent predictor of frailty (OR = 4.54, 95% CI: 1.52–13.53, *p* = 0.007).

For sarcopenia, hypopituitarism was not significantly different from the controls after adjustment (OR = 1.00, 95% CI: 0.30–3.33, *p* = 0.999), while acromegaly showed significantly lower odds of sarcopenia (OR = 0.09, 95% CI: 0.02–0.38, *p* = 0.001), consistent with the protective effect of GH/IGF-1 excess on muscle mass.

ANCOVA adjusting for age and sex confirmed that the hypopituitarism group had significantly higher TFI scores (β = 2.24, 95% CI: 0.71 to 3.77, *p* = 0.005) and significantly lower handgrip strength (β = −12.16, 95% CI: −17.36 to −6.96, *p* < 0.001) compared to controls. The acromegaly control difference in TFI scores was also significant (β = 1.62, 95% CI: 0.16 to 3.08, *p* = 0.030). No significant group differences were observed for SMI after adjustment.

Effect sizes for pairwise comparisons revealed very large effects for handgrip strength differences between hypopituitarism and both controls (Cohen’s d = −1.83, 95% CI: −2.48 to −1.17) and acromegaly (d = −1.43, 95% CI: −2.03 to −0.83), and large effects for TFI score differences (hypopituitarism vs. controls: d = 0.93, 95% CI: 0.36 to 1.51; acromegaly vs. controls: d = 0.76, 95% CI: 0.23 to 1.29). SMI showed a large effect for hypopituitarism vs. acromegaly (d = −0.80, 95% CI: −1.40 to −0.20) but negligible effects when comparing either group with controls.

## 4. Discussion

This study systematically evaluated frailty and sarcopenia in patients with hypopituitarism and acromegaly, using handgrip strength, the CT-derived skeletal muscle index (SMI), and the Tilburg Frailty Indicator, and compared these outcomes with healthy controls. In unadjusted analyses, our findings indicate that both pituitary disorder groups have higher rates of frailty, but sarcopenia exhibits distinct trends: acromegaly has a comparatively lower prevalence of sarcopenia, whereas hypopituitarism is associated with notable declines in skeletal muscle mass and strength [18]. Crucially, the relationship between hypopituitarism and frailty remained significant (OR = 5.24, *p* = 0.021) after controlling for age and sex, but the relationship between acromegaly and frailty displayed a borderline trend (OR = 3.00, *p* = 0.076).

These findings are in accordance with earlier research indicating that pituitary hormone shortages impair anabolic signaling, muscle regeneration, and protein synthesis, which in turn promotes sarcopenia and functional deterioration. On the other hand, skeletal muscles may be somewhat protected by excess GH/IGF-1 in acromegaly, which may account for the decreased incidence of sarcopenia despite frailty. The varied effects of pituitary hormone abnormalities on musculoskeletal health are highlighted by the distinct effects of hypopituitarism and acromegaly [33].

The results of Clegg and Hassan-Smith [2], which highlight the significance of the hypothalamic–pituitary axis in frailty etiology, are consistent with the increased prevalence of frailty in the acromegaly and hypopituitarism groups when compared to controls. Frailty may result from dysregulation within this axis, which can impact physiological reserves through androgens, IGF-1, and glucocorticoids. Furthermore, it has been demonstrated that frailty in the general population is correlated with IGF-1 and androgen levels [34,35]. These findings imply that clinical disorders that reflect opposing extremities of pituitary axis dysfunction may be associated with frailty. According to geriatric evaluations, frailty in acromegaly may be more noticeable than in controls and may be related to functional status and quality of life [36]. On the other hand, decreased muscle strength, exhaustion, and functional ability coexist with frailty in hypopituitarism [37].

GH insufficiency is especially important in hypopituitarism. GH-deficient people have poor quality of life, decreased bone mass, decreased muscle strength and exercise ability, and negative alterations in body composition [38,39]. Our hypopituitarism group’s noticeably reduced handgrip strength is in line with research on the effects of GH shortage on musculoskeletal function. According to Lissett et al. [40], IGF-1 levels in GH insufficiency can have a substantial impact on muscle mass and functional outcomes and may be influenced by the beginning of the disease. While GH replacement therapy has been demonstrated in certain studies to increase exercise capacity, adult GH insufficiency has been linked to decreased muscular strength and exercise tolerance [41,42]. Given the anabolic effects of the GH/IGF-1 axis, hypopituitarism’s functional decline and diminished muscle strength are probably caused by disruption of this route.

The hypopituitarism group’s CT-derived SMI values were lower than those of the acromegaly group, indicating that these patients’ muscle mass is also impacted. Although several factors, such as physical activity, dietary condition, and comorbidities, can affect muscle mass [43,44,45,46,47], the decreased SMI in hypopituitarism is consistent with the reported decreases in handgrip strength and supports the existence of sarcopenia. On the other hand, sarcopenia was uncommon in acromegaly (9%), probably due to comparatively retained muscle mass. However, research has revealed that despite intact muscle area, muscle quality and function may still be compromised in acromegaly due to fat infiltration or ectopic fat deposition, thereby impairing athletic performance [19,21,22]. Consequently, the significant frequency of frailty seen in acromegaly indicates that muscle mass alone cannot adequately account for functional limits.

The incidence of sarcopenia was high (54%) in the hypopituitarism group, which is consistent with reports showing that GH insufficiency raises the risk of sarcopenia [27,48,49,50]. However, in the adjusted logistic regression analysis, hypopituitarism was not significantly different from controls for sarcopenia risk (OR = 1.00, *p* = 0.999), while acromegaly demonstrated significantly lower odds (OR = 0.09, *p* = 0.001). This finding suggests that the apparent similarity in sarcopenia prevalence between the hypopituitarism and control groups may reflect shared risk factors (such as physical inactivity and diagnostic threshold sensitivity) rather than solely endocrine-driven pathology. Other anabolic axes, such as thyroid hormones and gonadal steroids, may also be impacted in addition to GH shortage. This might have a negative effect on muscle metabolism and regeneration ability by impairing mitochondrial activity and anabolic signaling [37,51,52,53]. The very high prevalence of sarcopenia in the control group (50%) was an unexpected finding that warrants careful interpretation. This likely reflects several factors: (1) EWGSOP2 cut-off thresholds derived from Western European populations may not be optimally calibrated for Turkish populations, as demonstrated by Bahat et al. [54]; (2) our use of mean-of-three grip strength values rather than the EWGSOP2-recommended best-of-three approach may have shifted some individuals below the cut-off; and (3) the opportunistic nature of CT availability may have introduced selection toward individuals with subclinical health concerns, The prevalence of sarcopenia in the general population is typically reported to be between 5% and 20% [30,54,55,56,57].

There are several limitations to this study that should be acknowledged. First, the statistical power of subgroup analysis may be limited due to the small sample size, and the wide confidence intervals in our logistic regression models reflect this constraint. Second, conclusive findings about the temporal relationships between frailty, sarcopenia, and pituitary diseases are not possible due to the cross-sectional methodology. Third, while CT imaging was used to measure muscle mass, additional physical performance metrics (such as gait speed or the Short Physical Performance Battery) could have provided a more comprehensive functional assessment. Fourth, the control group consisted of individuals who had undergone abdominal CT for nephrolithiasis evaluation; while none had documented comorbidities, the higher-than-expected sarcopenia prevalence (50%) suggests that EWGSOP2 cut-off thresholds may require population-specific calibration for Turkish cohorts. Fifth, physical activity level and detailed nutritional assessment (e.g., Mini Nutritional Assessment or dietary recall) were not collected, representing important unmeasured confounders. Sixth, handgrip strength was recorded as the average of three measurements rather than the EWGSOP2-recommended maximum value, which may have shifted some individuals below diagnostic cut-offs. Additionally, CT-based muscle measurements were performed by a single radiologist without formal intra- or inter-rater reliability assessment, which should be considered when interpreting SMI values. Seventh, subgroup analyses were not carried out to assess the distinct impacts of several hormone shortages (such as GH, thyroid-stimulating hormone, adrenocorticotropic hormone, and gonadotropins) on sarcopenia and frailty in hypopituitarism, and acromegaly patients were not stratified by disease activity status. Only one patient in the hypopituitarism group received growth hormone replacement therapy, limiting our ability to assess its protective effect.

Despite these limitations, the use of adjusted multivariable analyses enhances the internal validity of our results and underscores the potential role of pituitary hormones in frailty and muscle metabolism [58]. Additionally, a recent study reported that a flexiforce sensor integrated seat pan device exhibited high accuracy (*r* = 0.99) and excellent test-retest reliability (ICC = 0.99), effectively identifying poor posture in office workers and promoting self-correction through real-time biofeedback [59].

## 5. Conclusions

According to our results, frailty is more common in individuals with hypopituitarism and acromegaly in unadjusted analyses. The association between frailty and hypopituitarism remained significant after controlling for age and sex (OR = 5.24, *p* = 0.021), whereas acromegaly displayed a borderline tendency (OR = 3.00, *p* = 0.076). Sarcopenia is more prevalent in hypopituitarism, which is typified by a decline in handgrip strength and a lower SMI, than it is in acromegaly. However, the adjusted sarcopenia risk in hypopituitarism was not significantly different from controls (OR = 1.00, *p* = 0.999), while acromegaly showed a significantly lower sarcopenia risk (OR = 0.09, *p* = 0.001), consistent with the protective effect of GH/IGF-1 excess on muscle mass. Reductions in muscle mass alone may not be sufficient to explain functional impairment, as evidenced by the high frailty rates seen in acromegaly despite maintained muscle mass.

These results underline the potential significance of evaluating musculoskeletal health and functional status in the clinical management of patients with pituitary disorders and are broadly consistent with the viewpoint of Giustina et al. [60] regarding the role of the endocrine system in frailty and sarcopenia pathogenesis. Given the cross-sectional, clinic-based nature of this study and the limitations of our control group, these findings should be considered hypothesis-generating. Consideration of screening for frailty and sarcopenia in patients with pituitary disorders, particularly hypopituitarism, warrants further investigation. The effects of individual hormone deficiencies on muscle metabolism and frailty, the potential protective effects of hormone replacement therapies, and comprehensive assessments of muscle quality and functional status in acromegaly should all be investigated in future prospective studies with population-based control groups that are sufficiently powered.

## Figures and Tables

**Table 1:** Baseline Demographics of Hypopituitarism, Acromegaly, and Control Cohorts.

Variable	Control (*n* = 28)	Acromegaly (*n* = 32)	Hypopituitarism (*n* = 24)	*p*-Value
**Age (years),** **M** **± SD**	45.5 ± 12.6	45.8 ± 12.0	45.5 ± 16.8	0.99
**Gender, *n* (%)**				0.29
Male	15 (54)	11 (34)	9 (37)	
Female	13 (46)	21 (66)	15 (63)	
**Marital status, *n* (%)**				0.009
Married	26 (93)	18 (58)	16 (67)	
Single	2 (7)	13 (42)	8 (33)	
**Education level, *n* (%)**				0.35
Primary school	14 (50)	19 (61)	13 (54)	
High school	8 (29)	8 (26)	10 (42)	
University	6 (21)	4 (13)	1 (4)	
**Disease duration (years),** **M** **± SD**	-	10.1 ± 6.6	10.2 ± 6.6	0.54

**Table 2:** Treatment Profiles and Comorbid Conditions in Acromegaly and Hypopituitarism Groups.

Variable	Acromegaly (*n* = 32)	Hypopituitarism (*n* = 24)	*p*-Value
**Treatment History**			
Pituitary surgery, *n* (%)	28 (87.5)	12 (50.0)	0.003
Radiotherapy, *n* (%)	4 (12.5)	2 (8.3)	0.68
**Comorbidities**			
Any comorbidity, *n* (%)	24 (75.0)	14 (58.3)	0.19
Hypertension, *n* (%)	11 (34.4)	8 (33.3)	0.93
Diabetes mellitus, *n* (%)	10 (31.3)	5 (20.8)	0.38
Ischemic heart disease, *n* (%)	2 (6.3)	5 (20.8)	0.12

**Table 3:** Prevalence of Frailty and Sarcopenia and Measures of Muscle Strength Across Pituitary Disorder Cohorts.

Variable	Control (*n* = 28)	Acromegaly (*n* = 32)	Hypopituitarism (*n* = 24)	*p*-Value
**Frailty**				
Frailty status, *n* (%)	12 (43)	24 (75)	20 (83)	0.019
Tilburg Frailty Indicator Score, M ± SD	4.8 ± 2.9	7.0 ± 2.7	7.5 ± 2.8	0.015
**Muscle function**				
Muscle strength, M ± SD (95% CI)	36.4 ± 9.6 (30.8–41.9)	37.5 ± 14.3 (32.2–42.9)	20.8 ± 7.4 (17.6–23.9)	<0.001
SMI (cm^2^/m^2^), M ± SD (95% CI)	52.3 ± 13.7 (44.4–60.2)	53.0 ± 8.9 (49.6–56.4)	45.1 ± 11.3 (39.6–50.5)	0.04
**Sarcopenia**				
Sarcopenia status, *n* (%)	14 (50)	3 (9)	13 (54)	<0.001

## Data Availability

The data utilized in this study were prospectively collected from hospital information systems and patient medical records following approval by the Institutional Ethics Committee. Due to patient confidentiality and institutional policies, the dataset cannot be made publicly available. However, anonymized data relevant to this research can be made accessible by the corresponding author upon reasonable request, subject to obtaining additional ethical approval where necessary. Requests should be directed to the corresponding author via email at saydin1@hotmail.com.

## Abbreviations

The following abbreviations are used in this manuscript:

CT	Computed tomography
SMI	Skeletal muscle mass index
IGF	Insulin-like growth factor
GH	Growth hormone
